# BPA and Reproductive Health: Reviewing the Current State of the Science

**DOI:** 10.1289/ehp.122-A223

**Published:** 2014-08-01

**Authors:** Julia R. Barrett

**Affiliations:** Julia R. Barrett, MS, ELS, a Madison, WI–based science writer and editor, has written for *EHP* since 1996. She is a member of the National Association of Science Writers and the Board of Editors in the Life Sciences.

In 2006 a panel of experts reviewed the literature to that point on potential health effects arising from exposure to bisphenol A (BPA), a high-production-volume chemical that is broadly detectable in the environment as well as in most people’s bodies in developed countries.^1^ A new review takes stock of the knowledge gained since then, focusing on potential reproductive health effects while also considering new and lingering questions.^2^

BPA is a component of polycarbonate plastics and epoxies, and is used in products ranging from food and beverage containers to thermal-paper receipts. It has been found to leach from these products to varying degrees, imparting chronic, low-level exposures via dermal, respiratory, and oral routes.^1^^,^^3^ Such exposures were thought to be within the current reference dose set by the U.S. Environmental Protection Agency, but investigations conducted since that limit was set have suggested adverse health effects may occur at much lower doses.^1^^,^^3^

**Figure:**
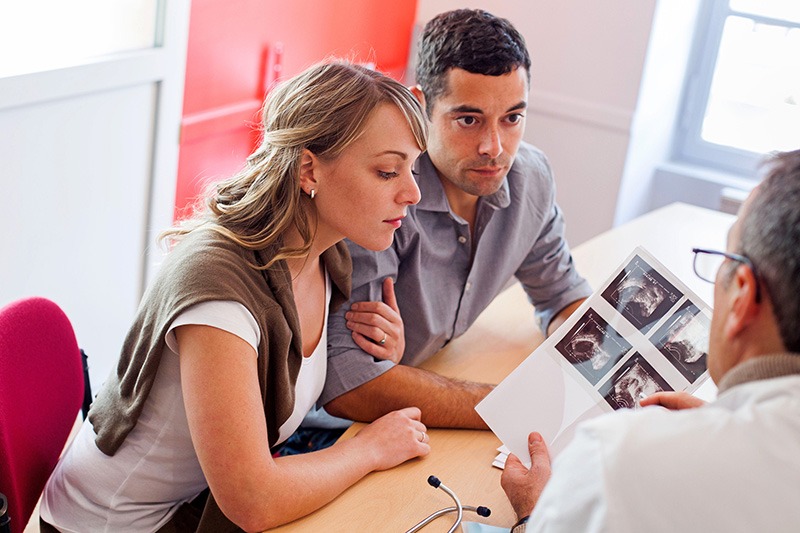
Aside from ovarian toxicity, the evidence for human reproductive effects of BPA is limited or inconclusive, according to a new review. © Burger/phanie/Phanie Sarl/Corbis

Furthermore, in contrast to early assumptions that BPA is an extremely weak estrogenic compound, recent investigations indicate that in some cases low-level BPA can stimulate cellular responses with a potency equal to estradiol (natural estrogen).^4^ In addition to genomic estrogen receptors, BPA binds to several other receptors that are involved in its various actions. Biomonitoring data indicate that most people are exposed to levels of BPA that are predicted to be biologically active.^1^^,^^3^

The current review draws on laboratory data and human studies published between 2007 and 2013.^2^ It takes a structured approach to assessing the wide-ranging body of intricate research. “I think the reason why the studies are so complicated is because BPA is very complex,” says coauthor Jodi Anne Flaws, a professor of comparative biosciences at the University of Illinois at Urbana–Champaign. “It probably has different mechanisms of action, in different tissues, in different species, at different doses, and at different developmental windows of exposure.”

The review encompasses both high- and low-dose studies involving pre-, neo-, and postnatal exposures and numerous end points. The consolidated data were deemed “strong” if multiple studies in multiple species indicated similar BPA-associated effects, “limited” if some but not most studies indicated a similar end point or if studies disagreed across species, or “inconclusive” if studies were limited in number or had only been done in one species or *in vitro.*

The authors concluded that strong evidence exists that BPA is an ovarian toxicant in both animals and humans and a uterine and prostate toxicant in animals. They concluded that evidence was limited for effects on the number of offspring, birth weight, and length of gestation; human studies were inconsistent, but animal studies were somewhat stronger. Evidence was also limited for BPA as a testicular toxicant and a factor in impaired embryo implantation in humans. Insufficient information is available to draw conclusions about BPA and effects on the oviduct, placenta, and pubertal development.

“The comparison of data from human studies with that of animal studies where possible is an important contribution and is a major strength of the manuscript,” says Beverly Rubin, an associate professor of integrative physiology and pathobiology at Tufts University. “The review provides an excellent resource for investigators in the field as well as those wanting to learn about BPA’s potential impacts on reproduction that—as illustrated here—are widespread, can occur at many different levels, and can be profoundly influenced by the window and the dose of exposure.” Rubin was not involved in the review.

The authors recommend that future studies emphasize critical periods during development and use continuous BPA exposure measures and doses that better reflect actual human exposure. Studies should also recognize potential interactions with coexisting factors, and differentiate between permanent and transient effects. Johanna Rochester, a research associate at the Endocrine Disruption Exchange who was not involved with the review, also suggests weighing how well studies are designed and executed, which she felt was a potential limitation in this otherwise strong work.

“One of the things that I think is really important with these systematic-type reviews is looking at study quality,” Rochester says. She would welcome further systematic reviews like the current one, saying, “If you can see *in vivo*, *in vitro*, and human results all presented together, showing the mechanisms, showing the relations between the doses, it’s much easier to get the bigger picture.”
